# Estimating the Reliability and Stability of Cognitive Processes Contributing to Responses on the Implicit Association Test

**DOI:** 10.1177/01461672231171256

**Published:** 2023-05-19

**Authors:** Jacob Elder, Liz Wilson, Jimmy Calanchini

**Affiliations:** 1 University of California, Riverside, USA

**Keywords:** intergroup bias, Implicit Association Test, racism, formal modeling, measurement reliability

## Abstract

Implicit measures were initially assumed to assess stable individual differences, but other perspectives posit that they reflect context-dependent processes. This pre-registered research investigates whether the processes contributing to responses on the race Implicit Association Test are temporally stable and reliably measured using multinomial processing tree modeling. We applied two models—the Quad model and the Process Dissociation Procedure—to six datasets (*N* = 2,036), each collected over two occasions, examined the within-measurement reliability and between-measurement stability of model parameters, and meta-analyzed the results. Parameters reflecting accuracy-oriented processes demonstrate adequate stability and reliability, which suggests these processes are relatively stable within individuals. Parameters reflecting evaluative associations demonstrate poor stability but modest reliability, which suggests that associations are either context-dependent or stable but noisily measured. These findings suggest that processes contributing to racial bias on implicit measures differ in temporal stability, which has practical implications for predicting behavior using the Implicit Association Test.

Implicit measures were initially assumed to assess stable individual differences that reflect durable associations stored in memory (Fazio et al., 1995; Greenwald et al., 1998; Wilson et al., 2000).^1^ However, responses on implicit measures often demonstrate low temporal stability despite adequate reliability within measurement occasion (Bar-Anan & Nosek, 2014; Cunningham et al., 2001; Gawronski et al., 2017; Lai & Wilson, 2021). This pattern of findings begs the question: To what extent are the underlying processes that contribute to responses on implicit measures stable within individuals? We rely on multinomial processing tree (MPT) models to disentangle the joint contributions of multiple cognitive processes to responses on the race Implicit Association Test (IAT: Greenwald et al., 1998), and we examine within-measurement reliability and between-measurement stability of parameters assumed to correspond to those latent processes. In doing so, we provide insight into the extent to which the cognitive processes that contribute to responses on the IAT are stable within individuals.

A variety of social cognitive theories assume that implicit measures primarily assess mental associations^2^ between target categories (e.g., “ingroup”) and attributes (e.g., “good”) that are stored in memory and persist over time (e.g., Fazio, 1990, 2007; Petty et al., 2007; Strack & Deutsch, 2004). Indeed, implicit measures were developed with operating conditions (e.g., short response windows) intended to facilitate the expression of associations by minimizing the expression of cognitive processes that may vary as a function of motivation, opportunity, or other contextual factors (Fazio et al., 1995; Greenwald et al., 1998). To the extent that implicit measures assess associations that are stable and enduring within persons, then responses on implicit measures should be expected to predict individual behaviors. Several meta-analyses have tested this assumption, and they estimated small-to-moderate relationships between the IAT and behavioral outcomes (Greenwald et al., 2009; Kurdi et al., 2019; Oswald et al., 2013). Like any other measurement tool, implicit measures must assess the intended construct reliably to effectively predict behaviors and other individual differences (Kanyongo et al., 2007; Loken & Gelman, 2017; Schmidt & Hunter, 1996). However, the IAT, in particular, has been criticized as a noisy measure (Blanton et al., 2009; Schimmack, 2021) due to its low retest stability across measurement occasions (Bar-Anan & Nosek, 2014; Cunningham et al., 2001; Gawronski et al., 2017; Lai & Wilson, 2021). To some degree, these criticisms depend on the assumption that the construct assessed by implicit measures is a stable individual difference—an assumption that is not universally accepted. For example, the Bias of Crowds (Payne et al., 2017) proposes that variance in responses on implicit measures is better explained by differences in situations and contexts than by differences between people. This contextual perspective dovetails with constructivist attitude theories (e.g., Conrey & Smith, 2007; Schwarz, 2007), which propose that responses on attitude measures (implicit or otherwise) do not reflect anything stable within individuals but, instead, reflect evaluations that are constructed on-the-spot based on information that is momentarily accessible—either in the mind or in the environment. To the extent that responses on implicit measures reflect constructs that are situationally dependent, then low retest stability across measurement occasions is unsurprising.

The unresolved debate over whether implicit measures assess something stable versus context-dependent largely depends on the assumptions that responses on implicit measures are relatively process-pure and primarily reflect the influence of mental associations. However, research using MPT models (Riefer & Batchelder, 1988) indicates that multiple cognitive processes jointly contribute to responses on implicit measures (Calanchini, 2020; Hütter & Klauer, 2016). Responses on implicit measures are traditionally quantified using summary statistics (e.g., Greenwald et al., 2003), but summaries can only provide limited insight when multiple processes influence responses. Thus, the extent to which responses on implicit measures reflect some processes that are stable within individuals and other processes that are not remains an open question. Compared with traditional summary statistics, MPT models are well-positioned to provide relatively more theoretically precise and statistically rigorous insight into the cognitive processes that contribute to responses on implicit measures.

In this research, we examine the extent to which the cognitive processes that contribute to responses on the race IAT reflect stable individual differences versus context-dependent processes. As an analog to how we conceptualize stability, people exhibit stability of personality traits across time (Caspi & Bem, 1990; Caspi et al., 1989; Roberts et al., 2008), such that they often maintain some degree of relative rank-ordering of behavior, regardless of average changes in the personality trait or behavior over time (i.e., the average may change, but the ordering is consistent). In the same way, we assume that some cognitive processes that contribute to responses on the IAT may be more or less stable across time. To examine stability in these processes, we examined the consistency of MPT parameters estimated from IATs administered across two measurement occasions. In psychometrics, retest stability metrics are used to parse between-individual variability in responses that represent “true” scores from within-individual variability that represents measurement error. However, within-individual variability across measurement occasions does not necessarily reflect measurement error if the underlying construct is context-dependent (Röseler et al., 2020; Steyer et al., 1992; Zuckerman, 1983). To determine whether low consistency across measurement occasion reflects instability in the underlying process versus measurement error, we must first establish whether the measure is reliable within measurement occasions. To help shed light on the extent to which IAT responses reflect within-individual variability versus error across measurement occasions, we also estimated the reliability of MPT parameters within each measurement occasion using parameter recovery. In summary, we establish within-measurement reliability using parameter recovery measurement, and we establish between-measurement stability using retest consistency measurement.

In this manuscript, we adopt the following terminology to distinguish between the tests we perform and the inferences we draw from those tests about the underlying constructs. We assess *consistency* (rather than absolute agreement, which accounts for systematic differences across timepoints) in parameter estimates across measurement occasions using intra-class correlations (ICCs) to draw inferences about the *stability* with which the constructs reflected in the model parameters can be measured. We also assess the *recoverability* of parameters within measurement occasions using parameter recovery to draw inferences about how *reliably* the constructs are measured by the model parameters.

Taken together, the analytic approach we adopt in the present research consists of two primary sets of analyses. To assess within-measurement reliability, we simulated data to determine the extent to which each MPT parameter can be reliably recovered. To assess between-measurement stability, we estimated the consistency of MPT parameters across occasions using ICCs. We will interpret parameters that demonstrate acceptable consistency and recoverability to reflect reliably measured and stable cognitive processes (i.e., trait individual differences), and interpret parameters that demonstrate poor consistency but acceptable recoverability to reflect reliably measured but unstable processes (i.e., time-dependent states). Parameters that demonstrate poor consistency and recoverability are unstable and unreliably measured, and therefore unlikely to be valid measures for individual-level inference. To increase the validity of our findings, we repeat this procedure across six independent datasets and meta-analyze the results. Moreover, to further maximize the validity of our findings, we apply two different MPT models (e.g., the Quad model and the Process Dissociation Procedure) to each dataset and look for consistent patterns of results in conceptually analogous MPT parameters.

## Method

### Study Selection

We relied on one dataset collected in our lab, and five other datasets from other sources. All six datasets consisted of data from the race IAT administered to participants on two measurement occasions. Sample sizes range from *n* = 32 to 1,240 participants, and intervals between measurement occasions range from a few minutes to 2 years (Table 1). Most of the data were collected online, so we do not have information about the physical locations in which the two measures were completed. Consequently, our analyses are well-positioned to provide insight into the extent to which processes reflect stable individual differences, but they provide insight only into the temporal dimension of context dependence.

**Table 1.** Description of Datasets

Source	*N*	Approximate time interval
Project Implicit (2020)	1,240	One browser session^a^
Wilson & Calanchini (2022)	105	24–48 hr
Lai et al. (2016) (Study 1)	80	1–4 days
Lai et al. (2016) (Study 2)	463	1–4 days
Gawronski et al. (2017)	116	1 month
Forscher et al. (2017)	32	2 years

*Note.* IAT = Implicit Association Test.

a The Project Implicit (2020) dataset consists of participants who completed two race IATs within the same browser session, but it does not record the time interval between IATs.

The present research was approved by the Institutional Review Board of the University of California Riverside #HS 20-278. Because this research relies on existing datasets, we did not conduct power analyses or determine sample sizes based on the present research questions. Similarly, these datasets may have included other manipulations or measures that are not relevant to our research questions, so we do not report or analyze those here. We describe our exclusion criteria below. Unless otherwise noted, all hypotheses and analyses were pre-registered. Pre-registrations, code, and data from Wilson & Calanchini (2022), Forscher et al. (2017), and Project Implicit (2020) are available at https://osf.io/qgvz3/?view_only=56aaa617de3545c297a5a1ce35b79ed2. Data from Lai et al. (2016) are available at https://osf.io/dbtns/. Data from Gawronski et al. (2017) are available at https://osf.io/792qj/.

### The Race IAT

All studies relied on the race IAT, which consists of stimuli reflecting two target categories (Black, White) and two attribute categories (good, bad^3^). The IAT proceeds in seven blocks, with the first block consisting of 20 practice trials in which participants categorize White stimuli and Black stimuli using two computer keys. The second block consists of 20 practice trials in which participants categorize good words and bad words. In the third and fourth critical blocks, the stimuli and response keys are combined, such that participants complete a total of 60 trials in which they respond to White and good stimuli with one response key, and to Black and bad stimuli with the other response key. The fifth block consists only of Black and White stimuli, and participants complete 20 practice trials with the response mapping reversed relative to the mapping in the previous blocks. In the sixth and seventh critical blocks, participants completed a total of 60 trials in which they respond to White and bad stimuli with one response key, and to Black and good stimuli with the other response key. Both of the Lai et al. (2016) datasets slightly deviated from this task structure. In these studies, participants completed an abbreviated version of the IAT with five blocks instead of seven blocks. Rather than four critical blocks, the abbreviated version consisted of two critical blocks of 32 trials each.

### Data Pre-Processing

We excluded participants who did not complete all IAT critical trials at both measurement occasions from analysis. In addition, we excluded participants who demonstrated IAT error rates exceeding 50%, which corresponds to random responding.

From the Gawronski et al. (2017) dataset, four participants’ responses were unable to be matched between measurement occasions due to an error in subject identifiers. From the Wilson and Calanchini (2022) dataset, 27 participants were excluded from analysis due to not fully completing the IAT at both measurement occasions. We examined a subset of respondents in the Project Implicit (2020) dataset and excluded participants who did not fully complete two separate IATs within the same browser session. After this exclusion, 12 additional participants in the Project Implicit (2020) dataset were removed for error rates exceeding 50%. After applying these exclusion criteria, we were left with the final sample sizes reported in Table 1.

### Multinomial Processing Tree Models

Responses on the IAT are traditionally quantified according to the D-scoring algorithm (Greenwald et al., 2003), which is a summary statistic that reflects the standardized difference between participants’ response latencies to one block of trials (e.g., when White stimuli share a response key with good attributes) versus another block of trials (e.g., when Black stimuli share a response key with good attributes). In the context of the race IAT, D-scores are interpreted such that values greater than zero are assumed to reflect relatively more positive evaluations of White people, and values less than zero are assumed to reflect relatively more positive evaluations of Black people. However, operationalizing responses on the IAT in terms of a relative summary statistic is theoretically imprecise: for example, differences in D-scores between experimental conditions may indicate that responses on the IAT are sensitive to manipulation but do not provide insight into which cognitive process or processes were influenced by the manipulation. In contrast, MPT modeling (Riefer & Batchelder, 1988) provides greater theoretical precision than do D-scores by quantifying the joint contributions of multiple cognitive processes to responses.

MPT models belong to a class of formal mathematical models that link latent processes to observable responses on tasks like the IAT (Batchelder & Riefer, 1999). MPT models are tailored to specific experimental paradigms that provide frequency data (e.g., number of correct and incorrect responses), and specify the number, nature, and composition of cognitive processes thought to contribute to responses in the paradigm (Hütter & Klauer, 2016). In creating MPT models, researchers must make theoretically grounded decisions about the specific manner in which multiple cognitive processes produce responses in each task condition. In this way, MPT models are mathematical instantiations of psychological theory packaged in a well-defined form.

An MPT model consists of parameters that correspond to latent cognitive processes, and the proposed interplay of these processes can be illustrated in a processing tree that consists of a root with multiple branches, with each branch corresponding to the success or failure of a process or series of processes. Each process is conditional upon the preceding process. The model estimates parameter values that most closely approximate participants’ observed responses across task conditions, and these parameter estimates are interpreted as probabilities that each cognitive process influenced participants’ responses.

In this research, we relied on two well-validated MPT models that have frequently been applied to the IAT: the quadruple process model (Quad model; Conrey et al., 2005) and the process dissociation procedure (PDP; Payne, 2001). The two models share a common dual-process perspective on implicit social cognition but differ in the number of processes proposed to influence responses, as well as in assumptions about the qualitative nature of those processes. In the present research, we applied both MPT models to the same IAT data, which not only provides a conceptual replication of our tests across models but also prevents us from making inferences based on a single operationalization of the cognitive processes underlying IAT performance. We can draw relatively stronger conclusions from our data if the same pattern of results emerges from both models.

### The Quad Model

**Figure 1.**
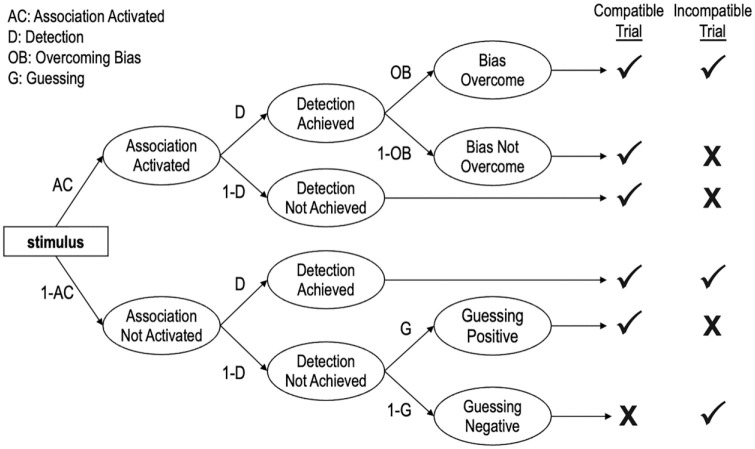
A Portion of the Quad Model. *Note.* The table on the right illustrates correct (✓) and incorrect (**X**) responses across different trial types.

The Quad model is depicted in Figure 1 and posits that observable responses in the IAT are produced by the joint influence of qualitatively distinct cognitive processes reflected in four model parameters (Conrey et al., 2005).

The activation of Associations parameter refers to the degree to which mental associations are activated when responding to a stimulus. All else being equal, the stronger the association between the target (e.g., White) and the attribute (e.g., good), the more likely the association is to be activated and drive responses in an association-consistent direction. We estimated two different Associations parameters: one reflects an association between White and good, and the other reflects an association between Black and bad. The Detection of correct responses parameter is conceptualized as an accuracy-oriented process, and it reflects the likelihood that the participant can discern the correct response. Sometimes activated associations conflict with the detected correct response. For example, on trials in which White faces appear and the categories “White” and “bad” share a response key, to the extent that a participant associates “White” with “good” then activated associations would conflict with the detected correct response. The Quad model proposes an Overcoming Bias parameter to resolve such a conflict between Associations and Detection. The Overcoming Bias parameter refers to an inhibitory process that prevents activated associations from influencing behaviors when they conflict with detected correct responses. Finally, the Guessing parameter does not represent a specific process, per se, but instead reflects the tendency to respond with “good” versus “bad” in the absence of influence from the Associations, Detection, and Overcoming Bias parameters.

### The Process Dissociation Procedure

**Figure 2.**
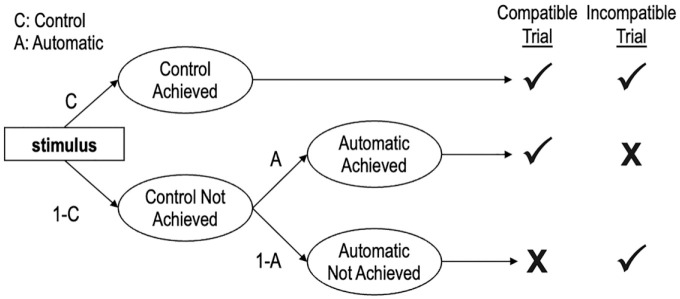
The Process Dissociation Procedure. *Note.* The table on the right depicts correct (✓) and incorrect (**X**) responses across different trial types.

The PDP is depicted in Figure 2 and posits that observable responses on the IAT are produced by the joint influence of qualitatively distinct cognitive processes reflected in two model parameters.^4^

The Automatic parameter refers to the degree to which associations between targets and attributes drive responses. Similar to how the Associations parameter of the Quad model is assumed to operate, the stronger the association between a target (e.g., White) and an attribute (e.g., good), the more likely the association is to be activated and drive responses in an association-consistent direction. We estimated two different Automatic parameters, one for White stimuli and another for Black stimuli. Importantly, though the Automatic parameters of the PDP and the Associations parameters of the Quad model are conceptually analogous, they are operationalized differently in each model. The Associations parameters of the Quad model assume a direction of compatibility, such that they are traditionally specified to reflect associations between White people and good attributes, and between Black people and bad attributes; consequently, larger parameter values reflect stronger links between White-good and Black-bad. In contrast, the Automatic parameters of the PDP do not assume a direction of compatibility: parameter values >0.5 reflect positive evaluations and values <0.5 reflect negative evaluations of each target group.

The Control parameter reflects the correct processing of stimuli and how well participants can distinguish between target concepts and attributes. The Control parameter in the PDP and the Detection parameter in the Quad model are conceptually analogous, in that both refer to accuracy-oriented cognitive processes. However, the two parameters differ in their specifications. In the PDP, the success of the Control parameter will always lead to a correct response, and the Automatic parameter can only influence responses in the absence of influence from the Control parameter. In contrast, in the Quad model, the success of the Detection parameter depends on the success of the Overcoming Bias parameter when the Associations parameter would produce a response that conflicts with Detection. We estimated four different Control parameters, one each for White, Black, good, and bad stimuli.

Because the PDP does not include a catch-all parameter like Guessing in the Quad model, the Automatic parameters of the PDP must be interpreted differently from the Associations parameters of the Quad model. Specifically, the influence of any cognitive processes that are not accounted for by the Control parameter is necessarily reflected in the Automatic parameter. The PDP assumes that the Control parameter will always drive responses, even if the Automatic parameter is also activated. In contrast, the Quad model assumes that either the Detection parameter or the Associations parameter can drive responses if both are activated, and the success or failure of the Overcoming Bias parameter determines whether Detection or Associations drive responses, respectively. Consequently, the Automatic parameter of the PDP can be interpreted to reflect a combination of the Associations, Overcoming Bias, and Guessing parameters of the Quad model.

### Interpretation of Processes

Because the Associations (in the Quad model) and Automatic (in the PDP) parameters are conceptualized to reflect associations between target groups (e.g., White) and evaluations (e.g., good), they would seem to most closely correspond to the construct that implicit measures are traditionally assumed to assess. If these parameters demonstrate fair consistency between measurement occasions and acceptable recoverability within measurement occasions, we will interpret them to reflect a reliable measure of stable individual differences. However, if they demonstrate poor consistency but acceptable recoverability, we will interpret them to reflect a reliable measure of a time-dependent process.

The other parameters reflected in the Quad model and PDP do not correspond as closely as do the Associations and Automatic parameters to the constructs that implicit measures are traditionally assumed to assess. Nevertheless, these other parameters are estimated from IAT responses, so investigating the extent to which they are reliable and stable across measurement occasions may still be informative. The Detection and Control parameters are conceptualized to reflect accuracy-oriented processes, and the Overcoming Bias parameter is conceptualized to reflect an inhibitory process. Guessing does not reflect a specific process, but instead reflects any processes that influence IAT responses that are not accounted for by the other model parameters, akin to residual error terms in structural equation modeling. We will interpret these parameters in the same way that we interpret the Associations and Automatic parameters: parameters that demonstrate fair consistency and acceptable recoverability reflect reliable measures of stable processes, and parameters that demonstrate poor consistency but acceptable recoverability reflect processes that are reliable measures of time-dependent processes. A parameter with poor recoverability is likely to also have poor consistency and/or contain substantial measurement error and, thus, should not be relied on for individual-level inference.

### MPT Estimation

To quantify the influence of each process specified in each MPT model, we implemented an approach that relies on hierarchical Bayesian estimation (Klauer, 2010). This approach assumes that individual-level parameters are drawn from a multivariate normal population distribution, thereby regularizing and stabilizing individual-level estimates (Ahn et al., 2017). We fitted all hierarchical MPT models using default priors in *TreeBUGS* (Heck et al., 2018) in R Programming Environment v.4.1.2., which draws posterior samples of the parameters using Markov chain Monte Carlo methods. We ensured sufficient parameter convergence of all models using a criterion of a Gelman-Rubin R-hat < 1.05 and a visual inspection of Gelman-Rubin trace plots.

### Pre-registered Analyses

#### Parameter Consistency Between Measurement Occasion

We assessed parameter consistency between measurement occasions using ICCs representing the ratio of intra- to inter-individual variance (Koo & Li, 2016; McGraw & Wong, 1996). We modeled two-way mixed effects ICCs, such that both participants and measurement occasions were treated as random effects sampled from a larger pool of people and time points. We focused on the relative ranking of participants across timepoints, rather than absolute agreement without error, and thus relied on consistency ICCs. Each ICC was conducted with only two timepoints, and we report ICCs with confidence intervals (CIs). We defined as ICC(3,1) according to Shrout and Fleiss (1979) convention,^5^ and estimated ICCs using the *irr* package 0.84.1 (Brueckl & Heuer, 2022).

#### Parameter Recovery Within Measurement Occasion

Parameter recovery is a method to investigate the extent to which a specific model configuration can reliably reproduce parameter estimates given a set of behavioral data (i.e., the model is identifiable). In doing so, parameter recovery provides us with insight into the extent to which a parameter reflects an estimate of the intended construct versus measurement error (Ballard et al., 2020; Shahar et al., 2019). The parameter recovery process consists of four steps. First, we estimate a set of model parameters from real participants’ responses (i.e., “original” parameters). Second, we simulate behavioral data based on the original parameters. Third, we fit the model to the simulated data to produce a new set of parameter estimates (i.e., “simulated” parameters). Fourth, and finally, we compare the simulated parameters to the original parameters. If a model’s parameters can be successfully recovered, there will be a tight correspondence between original and simulated parameters—which, in turn, provides a “ground truth” to establish the reliability of parameter estimation. As an analogy, parameter recovery in this context can be considered akin to a psychometric investigation of internal consistency, which may be more familiar to many readers. Internal consistency reflects the extent to which items on an inventory are correlated with one another and, thus, quantifies whether the measurement of a construct can be trusted. Similarly, parameter recovery reflects the extent to which a set of parameter values can generate behavior that reproduces the same parameters and, thus, quantifies whether the measurement of a parameter can be trusted.

We simulated behavioral data using the *rmultinorm* function in R based on the original Quad model and PDP parameters estimated from each dataset. The behavioral data corresponded to two choice outcomes—correct, incorrect—for each response category (i.e., responses to White, Black, pleasant, and unpleasant stimuli in compatible and incompatible IAT blocks). Then, we applied the Quad model and PDP to the simulated data and estimated new parameters. Finally, we calculated Pearson correlations between the original and recovered estimate of each parameter for each model for each dataset and time point.

### Meta-Analysis

To synthesize our findings across datasets, we performed random-effects meta-analyses using the *metafor* package 3.0-2 in R (Viechtbauer, 2010). For each parameter of each MPT model, we conducted one meta-analysis based on the consistency analyses, and another meta-analysis based on the recovery analyses. Whereas the consistency analyses necessarily reflected data from both measurement occasions (i.e., quantifying the extent to which responses at Time 1 correspond with responses at Time 2), the recovery analyses reflected data within each measurement occasion, thereby providing twice as many estimates in the recovery meta-analysis as in the consistency meta-analysis. Consequently, we modeled measurement occasion as a random factor nested within study in a multilevel meta-analysis of the recovery estimates.

We estimated the standard error of all ICCs using the Fisher *r*-to-*Z* transformation for ICC values (Chen et al., 2018). For the final reported meta-analytic estimates, we converted the estimates and their 95% CIs via a *Z*-to-*r* transformation. As inference criteria, we compare CIs to determine if meta-analytic estimates are significantly different from one another. For example, if the CIs of two parameters’ meta-analytic ICCs do not overlap (i.e., the ICC’s lower bound for one parameter is greater than the upper bound for another parameter), we will conclude that the two parameters’ ICCs are different from one another.

## Results of Pre-Registered Analyses

### Parameter Consistency Between Measurement Occasions

To estimate whether parameters are consistent between measurement occasions, we calculated ICCs for Quad and PDP parameters and meta-analyzed the results. Between-measurement occasion consistency is often interpreted using different criteria than is within-measurement occasion reliability (Matheson, 2019), given that changes may reflect changes in true scores or measurement error. We interpret ICCs according to the criteria proposed by Cicchetti and Sparrow (1981): <.40 is poor; .40 to .60 is fair; .60 to .75 is good; >.75 is excellent.

#### The Quad Model

**Figure 3.**
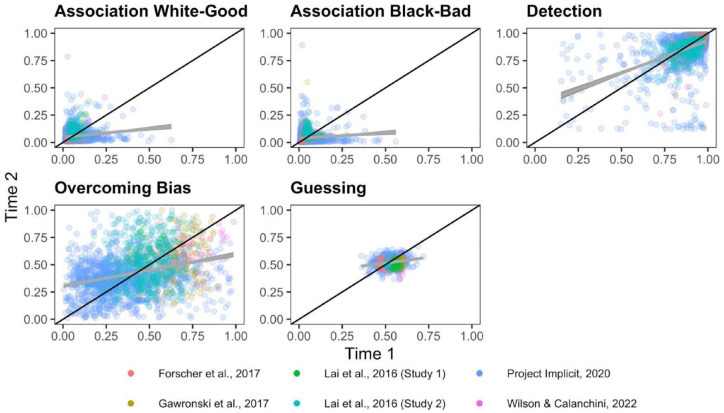
Scatterplots Depicting the Relationship Between Time 1 and Time 2 Quad Parameters. *Note.* The black diagonal line depicts perfect consistency between Time 1 and Time 2 and the gray line depicts best-fit slopes through the observed data.

**Figure 4.**
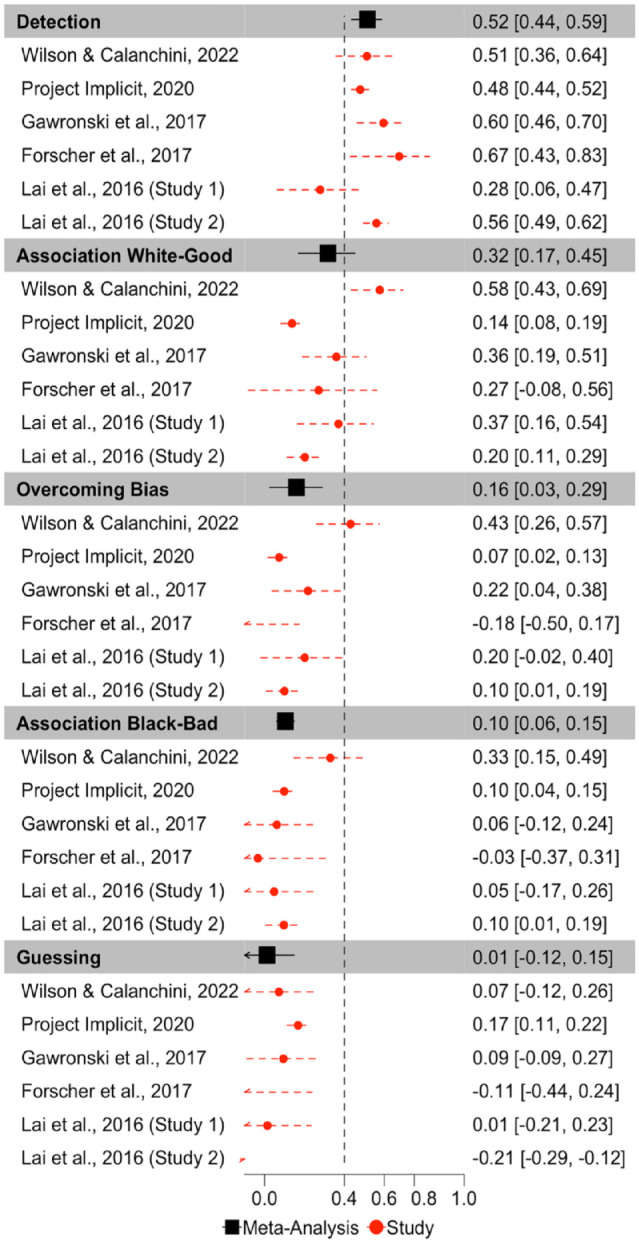
Forest Plot Depicting the Meta-Analytic and Study Specific Consistencies for the Quad Model, Depicted as Intraclass Correlations. *Note.* Point estimates and 95% confidence intervals are reported. Dashed vertical line reflects the threshold of acceptable consistency.

ICCs for each Quad parameter for each dataset are depicted in Figure 3. Meta-analytic results (Figure 4) indicate that Detection parameters were the most consistent across measurement occasions of all Quad parameters, and demonstrate fair consistency, ICC(3,1) = .515, 95% CI = [.436,.587], *p* < 001. The other parameters demonstrated poor consistencies: White-good Associations ICC(3,1) = .318, 95% CI = [.170, .452], *p* < .001; Black-bad Associations ICC(3,1) = .104, 95% CI = [.061, .147], *p* < .001; Overcoming Bias ICC(3,1) = .160, 95% CI = [.026, .289], *p* = .020; Guessing ICC(3,1) = .012, 95% CI = [−.124, 148], *p* = .863. The consistency of the Guessing parameter approaches 0 and its CI includes negative values, indicating that its within-subject variance exceeds its between-subject variance, and suggesting that Guessing reflects more noise than signal.

Inspection of CIs indicates that Detection exhibits higher consistency than do Black-bad Associations, Overcoming Bias, and Guessing, but does not differ from the consistency of White-good Associations. White-good Associations demonstrate higher consistency than Guessing, but does not differ from the consistency of Black-bad Associations or Overcoming Bias. Black-bad Associations, Overcoming Bias, and Guessing do not differ in their consistency.

There was substantial heterogeneity in consistency between studies for Detection (Q(5) = 13.717, *p* = .018, *I*^2^ = 67.626), Guessing (Q(5) = 50.089, *p* < .0001, *I*^2^ = 82.406), Overcoming Bias (Q(5) = 18.887, *p* = .002, *I*^2^ = 82.125), and White-good Associations (Q(5) = 32.507, *p* < .0001, *I*^2^ = 86.163), which suggests that these parameters varied in their ICCs across studies. However, there was minimal variation in Black-bad Associations ICCs across studies, Q(5) = 6.819, *p* = .235, *I*^2^ = 0.

#### The Process Dissociation Procedure

**Figure 5.**
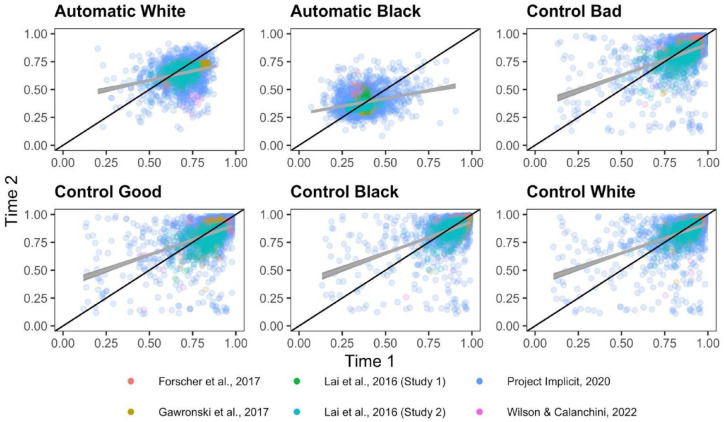
Scatterplots Depicting the Relationship Between Time 1 and Time 2 PDP Parameters. *Note.* The black diagonal line depicts perfect consistency between Time 1 and Time 2 and the gray line depicts best-fit slopes through the observed data. PDP = process dissociation procedure.

**Figure 6.**
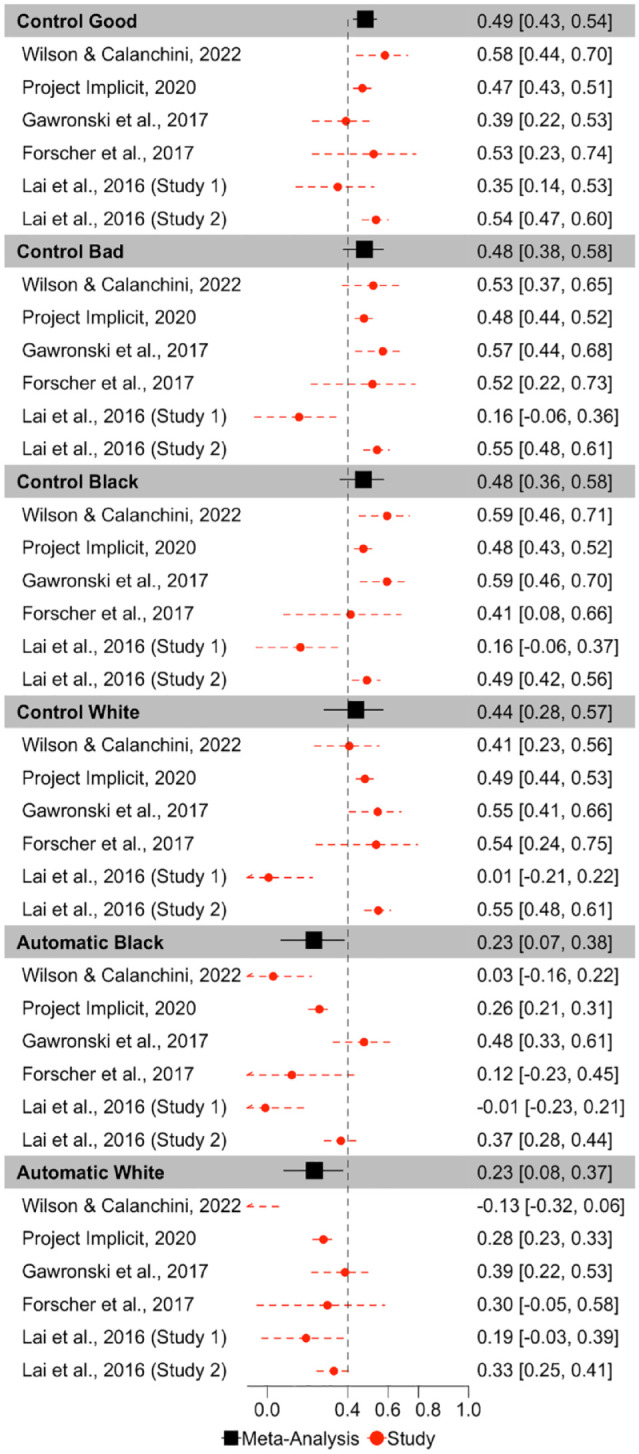
Forest Plot Depicting the Meta-Analytic and Study Specific Consistencies for the PDP, Depicted as Intraclass Correlations. *Note.* Point estimates and 95% confidence intervals are reported. Dashed vertical line reflects the threshold of acceptable reliability. PDP = process dissociation procedure.

ICCs for each PDP parameter for each dataset are depicted in Figure 5. Meta-analytic results (Figure 6) indicate that Control parameters were the most consistent across measurement occasions of all PDP parameters, exhibiting fair consistency: Control-good ICC(3,1) = .487, 95% CI = [.428, .578], *p* < 001; Control-bad ICC(3,1) = .483, 95% CI = [.377, .576], *p* < .001; Control-Black ICC(3,1) = .477, 95% CI = [.362, .578], *p* < .001; Control-White ICC(3,1) = .440, 95% CI = [.283, .574], *p* < .001. However, both Automatic parameters demonstrated poor consistency: Automatic-White ICC(3,1) = .233, 95% CI = [.082, .374], *p* = .003; Automatic-Black ICC(3,1) = .232, 95% CI = [.069, .382], *p* = .006.

Inspection of confidence intervals indicates that Control-good exhibits significantly higher consistency than do both of the Automatic parameters. Control-bad also exhibits significantly higher consistency than does Automatic-White but does not differ in consistency from Automatic-Black. The consistency of the other two Control parameters does not differ from the consistency of either of the Automatic parameters. None of the Control parameters differs from one another in terms of consistency, nor do the Automatic parameters differ from one another in terms of consistency.

There was substantial heterogeneity in consistency between-studies for Automatic-Black (Q(5) = 25.506, *p* = .0001, *I*^2^ = 88.608), Automatic-White (Q(5) = 21.914, *p* = .0005, *I*^2^ = 86.758), Control-Black (Q(5) = 15.738, *p* = .008, *I*^2^ = 83.326), Control-bad (Q(5) = 15.580, *p* < .0001, *I*^2^ = 80.419), Control-White (Q(5) = 26.757, *p* < .0001, *I*^2^ = 90.539). Control-good was the only parameter that did not significantly vary in terms of consistency across studies, Q(5) = 8.611, *p* = .126, *I*^2^ = 42.710.

### Parameter Recovery Within Measurement Occasions

To establish whether parameters are reliable within a measurement occasion, we performed parameter recovery. These analyses provide insight into whether the data-generating process for these parameters can reliably recover the same parameters, and therefore whether the process can be reliably measured—which, in turn, illuminates the extent to which stability between measurement occasions reflects within-individual variability versus measurement error. We considered parameter recovery to be acceptable if *r* > .70 (Nunnally, 1978; Nunnally & Bernstein, 1994; Palminteri et al., 2017).

#### The Quad Model

**Figure 7.**
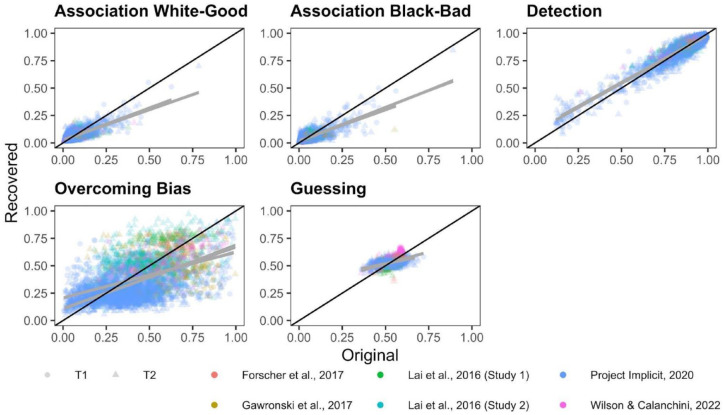
Scatterplots Depicting the Relationship Between the Original and Simulated Quad Parameters. *Note.* The black diagonal line depicts perfect recovery and the gray line depicts best-fit slopes through the observed data. Each study is depicted as a different color. Time 1 and Time 2 parameter recoveries were conducted separately and are depicted as different shapes.

**Figure 8.**
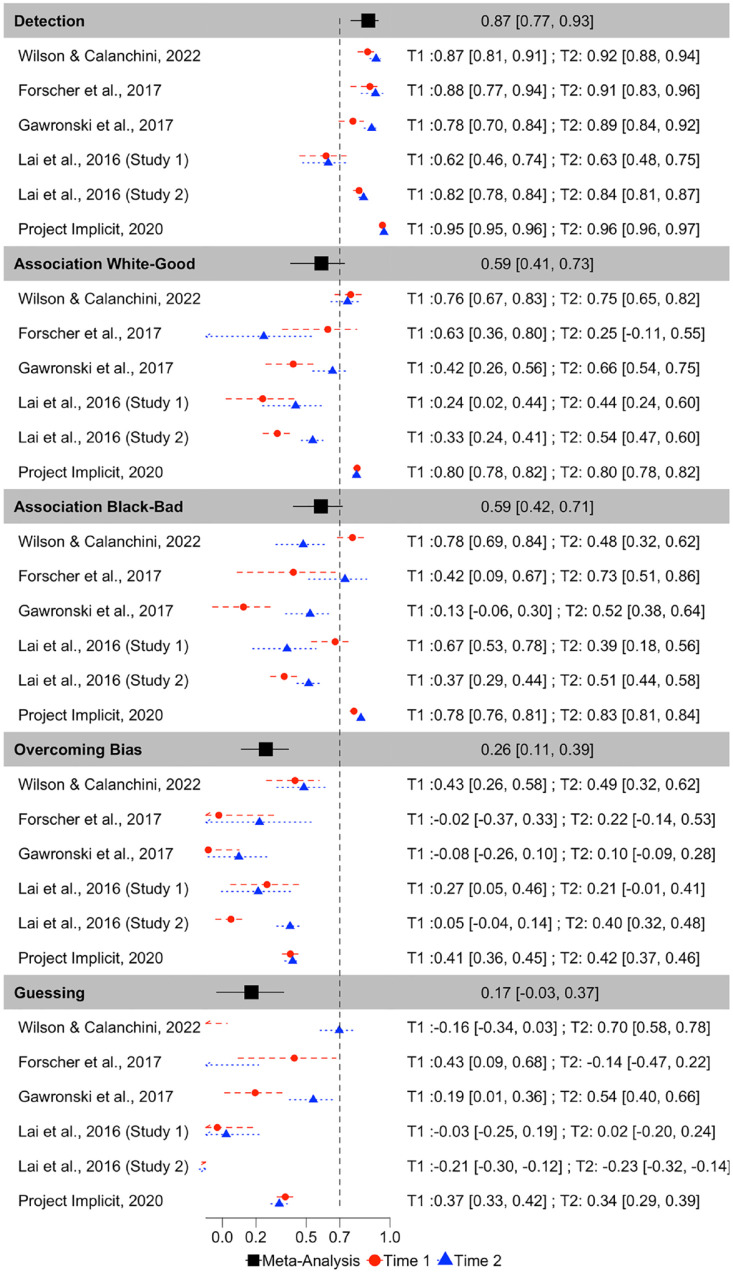
Forest Plot Depicting the Meta-Analytic and Study-Level (for Each Measurement Occasion) Recovery Estimates for Each Quad Parameter, in Terms of Pearson r Correlations. *Note.* Point estimates and 95% confidence intervals are reported. Dashed vertical line reflects the threshold of acceptable recoverability.

Correlations between original and recovered parameters for each Quad parameter are depicted in Figure 7. Meta-analytic results (Figure 8) indicate that Detection parameters were the most recoverable of all Quad parameters, and demonstrate acceptable recovery: *r* = .870, 95% CI = [.767, .929], *p* < 001. The other Quad parameters did not demonstrate acceptable recovery: White-good Associations *r* = .590, 95% CI = [.406, .728], *p* < .001; Black-bad Associations *r* = .588, 95% CI = [.423, .715], *p* < .001; Overcoming Bias *r* = .259, 95% CI = [.115, .394], *p* = .0006; Guessing *r* = .173, 95% CI = [−.035, .367], *p* = .102. That said, the Associations parameters both demonstrated modest recoverability that approached the threshold for acceptable, whereas Overcoming Bias and Guessing demonstrated unequivocally poor recovery that approached or included 0.

Inspection of CIs indicates that Detection exhibited significantly higher recoverability than do any of the other Quad parameters. Both Associations parameters also demonstrated significantly higher recoverability than the Overcoming Bias and Guessing parameters. The Associations parameters did not differ from one another in terms of recoverability, nor did the Overcoming Bias and Guessing parameters differ from one another in terms of recoverability.

There was substantial heterogeneity in recoverability between-studies for Detection (Q(11) = 600.942, *p* < .001, *I*^2^ = 97.878), Guessing (Q(11) = 329.556, *p* < .0001, *I*^2^ = 97.193), Overcoming Bias (Q(11) = 102.428, *p* < .0001, *I*^2^ = 91.857), White-good Associations (Q(11) = 416.096, *p* < .0001, *I*^2^ = 96.670), and Black-bad Associations (Q(11) = 372.995, *p* < .0001, *I*^2^ = 96.603). The amount of heterogeneity in recovery is surprising, given that recovery estimates should be relatively stable given a particular model configuration. We report exploratory analyses later in this manuscript in which we further probe this point.

#### The Process Dissociation Procedure

**Figure 9.**
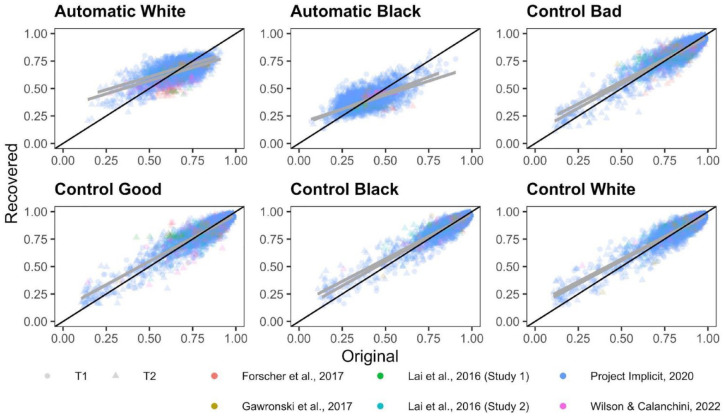
Scatterplots Depicting the Relationship Between the Original and Simulated PDP Parameters. *Note.* The black diagonal line depicts perfect recovery and the gray line depicts best-fit slopes through the observed data. Each study is depicted as a different color. Time 1 and Time 2 parameter recoveries were conducted separately and are depicted as different shapes. PDP = process dissociation procedure.

**Figure 10.**
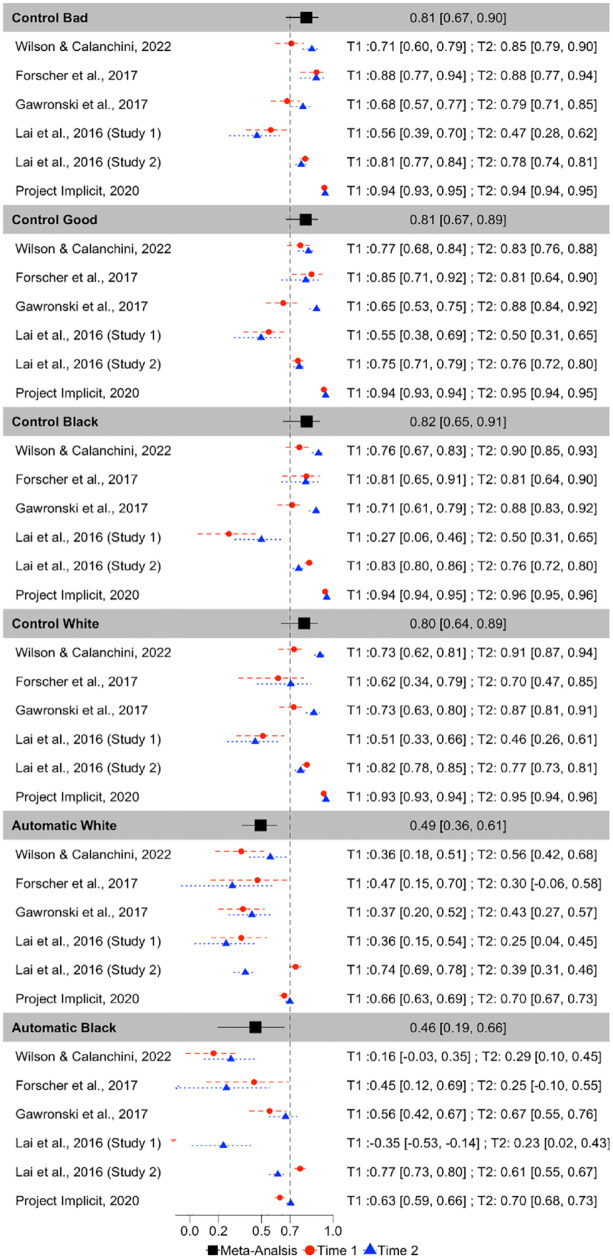
Forest Plot Depicting the Meta-Analytic and Study-Level (for Each Measurement Occasion) Recovery Estimates for Each PDP Parameter, in Terms of Pearson r Correlations. *Note.* Point estimates and 95% confidence intervals are reported. Dashed vertical line reflects the threshold of acceptable recoverability. PDP = process dissociation procedure.

Correlations between original and recovered parameters for each PDP parameter are depicted in Figure 9. Meta-analytic results (Figure 10) indicate that Control parameters were the most recoverable, with point estimates that all demonstrate acceptable recovery: Control-Black *r* = .816, 95% CI = [.653, .907], *p* < 001; Control-bad *r* = .813, 95% CI = [.671, .898], *p* < .001; Control-good *r* = .808, 95% CI = [.671, .892], *p* < .001; Control-White *r* = .798, 95% CI = [.638, .892], *p* < .001. The Automatic parameters demonstrated modest recoverability, but did not meet the a priori threshold of .70 for acceptable recoverability: Automatic-White *r* = .494, 95% CI = [.364, .606], *p* < .0001; Automatic-Black *r* = .458, 95% CI = [.195, .660], *p* < .0001.

Inspection of CIs indicates that all four Control parameters exhibited significantly higher recoverability than both of the Automatic parameters. The Control parameters did not differ from one another in terms of recoverability, nor did the Automatic parameters differ from one another in terms of recoverability.

There was substantial heterogeneity in recoverability between-studies for Automatic-Black (Q(11) = 236.075, *p* = .0001, *I*^2^ = 97.744), Automatic-White (Q(11) = 163.855, *p* < .0001, *I*^2^ = 93.730), Control-Black (Q(11) = 700.643, *p* < .0001, *I*^2^ = 98.460),, Control-good (Q(11) = 630.490, *p* < .0001, *I*^2^ = 97.833), Control-White (Q(11) = 598.092, *p* < .0001, *I*^2^ = 98.225), and Control-bad (Q(11) = 571.550, *p* < .0001, *I*^2^ = 97.893).

## Exploratory Analyses and Results

In addition to the pre-registered analyses reported above, we summarize below two exploratory analyses. These analyses aim to address additional questions that arose over the course of conducting the pre-registered analyses related to the repeatability of IAT responses across simulations, and to the moderating role of time between measurement occasions on parameter consistency.

### Repeatability of IAT Responses Across Simulations

Given the high heterogeneity in recoverability estimates identified across samples, we investigated the repeatability with which a given set of parameters can estimate similar response frequencies. Our exploratory analyses (which are described in full in the Supplement) examined repeatability in two ways: in terms of the relationships among multiple simulations of responses, and in terms of the relationship between simulated responses and participants’ original responses.

Supplemental Table 1 summarizes the repeatability among simulated response frequencies for the Quad model, and Supplemental Table 2 summarizes repeatability among simulated response frequencies for the PDP. Across both models, simulated responses were weakly-to-moderately associated with other simulated responses, whereas simulated responses were more strongly associated with participants’ original responses. Project Implicit is the largest sample and exhibits the strongest repeatability estimates, which potentially reflects more accurate population-level estimates that help to regularize individual-level estimates at larger sample sizes.

### Time as a Moderator of Consistency Between Measurement Occasion

The amount of time elapsed between measurement occasions may reasonably be expected to moderate the extent of parameter consistency, such that shorter intervals between measurement occasions would be related to higher consistency. Based on the time intervals between measurement occasions listed in Table 1, we conducted a series of exploratory orthogonal polynomial contrasts to examine the relationship between time between measurement and consistency for each MPT parameter.

For the Quad model, there was a marginal trend of linear moderation, and a trend of quadratic moderation, on consistency by the categorical order of time interval length for Overcoming Bias, Q_M_(4) = 18.714, *p* = .001; β_Linear_ = −.239, SE_Linear_ = 0.126, p_Linear_ = 0.059; β_Quadratic_ = −.303, SE_Quadratic_ = .109, p_Quadratic_ = .006, which suggests that Overcoming Bias may initially decrease and then increase in consistency over time. Categorical order of time length did not moderate consistency for the other Quad parameters, nor for any of the PDP parameters. Thus, there does not appear to be much evidence that time between IAT measurement occasions moderates the consistency of MPT parameters.

## Discussion

The present research investigates the extent to which the processes that contribute to responses on the race IAT are stable within individuals over time and can be reliably measured. Aligning with calls for more formal modeling in psychological science (Robinaugh et al., 2021; Smaldino, 2020), we investigated these questions using the Quad model and PDP to concretely specify our theoretical assumptions, and meta-analyzed our findings across six datasets collected by multiple laboratories to increase the validity of our findings.

We found that parameters reflecting two accuracy-oriented processes (i.e., responses that correctly identify stimulus)—Detection in the Quad model and Control in the PDP—generally demonstrated fair consistency between measurement occasions and acceptable recoverability within measurement occasion. This pattern of results suggests that both parameters reflect relatively stable individual differences. In contrast, parameters reflecting associations between target groups and attributes—Associations in the Quad model and Automatic in the PDP—did not meet our a priori thresholds for fair consistency or acceptable recoverability. One interpretation of this pattern of results is that the Associations and Automatic parameters do not reflect stable individual differences. However, because these two parameters demonstrate what could be reasonably characterized as modest recoverability, with *r*s > .58 for Associations, and *r*s >.45 for Automatic, we cannot rule out the alternative possibility that the Associations and Automatic parameters reflect relatively noisily-measured individual differences. Finally, the Overcoming Bias and Guessing parameters in the Quad model both demonstrated unequivocally poor consistency and recoverability, suggesting that these parameters are unstable, likely contain a large degree of measurement error in their estimation, and should not be interpreted as individual-level estimates.

### Theoretical Implications for Implicit Measures

Our findings that the accuracy-oriented processes that contribute to responses on the IAT—Detection and Control—are reliable within measurement occasions and relatively stable over time is consistent with literature suggesting that executive functions reflect stable individual differences (Beck et al., 2011; Miyake & Friedman, 2012; Paap & Sawi, 2016; Willoughby & Blair, 2011). This pattern of results also dovetails with previous research linking the Control parameter of the PDP estimated from the IAT with the executive functions of working memory updating and task shifting (Ito et al., 2015). That said, Overcoming Bias is conceptualized as an inhibitory process, which also situates it among the constellation of executive functions—and, as such, should be expected to reflect a stable individual difference. However, the present research indicates that the Overcoming Bias parameter is neither stable nor reliable. Future research is necessary to clarify why some executive function-related parameters, such as Detection and Control, are reliable and stable, but other executive function-related parameters, such as Overcoming Bias, are unreliable and unstable.

In contrast to the pattern of results we observe for the Detection and Control parameters, our findings that Associations and Automatic parameters are relatively less stable over time would seem to pose a challenge for the perspective that responses on implicit measures reflect associations between target groups and attributes that are durably stored in memory (Greenwald et al., 1998; Petty et al., 2008). Though we cannot rule out the possibility that these parameters reflect relatively stable but noisily-measured individual differences, their low consistency and modest recoverability support a context-dependent perspective on implicit social cognition (Conrey & Smith, 2007; Payne et al., 2017; Schwarz, 2007). Context can be operationalized in a variety of ways—including physical spaces, geographical areas, social situations, internal states, or specific times—and our data can only speak to the time-dependence of model parameters. To date, much of the evidence investigating context-dependent perspectives in implicit social cognition has focused on physical space (Hannay & Payne, 2022; Ofosu et al., 2019; Vuletich & Payne, 2019). However, most of the data reflected in the present research was collected over the internet and, thus, we have little information about the physical spaces in which participants completed these IATs. Future research is needed to discern whether Associations and Automatic parameters reflect context-dependent versus stable but noisily-measured constructs (Carpenter et al., 2022; Connor & Evers, 2020), and tightly controlled measurement settings may provide deeper insight into situational features related to the context-dependence of these constructs.

We relied on two qualitatively distinct MPTs in the present research with an eye toward the validity of our findings. To the extent that we find a pattern of results across conceptually analogous parameters in each MPT, then we can have relatively high confidence that our findings do not reflect idiosyncrasies of our modeling choices. Indeed, we found a very similar pattern of results across the Detection parameter of the Quad model and the Control parameters of the PDP in terms of both consistency and recoverability. However, the Associations parameters of the Quad model were descriptively more recoverable than were the Automatic parameters of the PDP. One possible explanation for this divergence between Associations and Automatic parameters is that the Quad model includes the Guessing parameter as a catch-all, of sorts, that reflects the influence of any other processes is not accounted for in the Associations, Detection, or Overcoming Bias parameters. Because the PDP does not include a Guessing parameter, Automatic parameters necessarily reflect the influence of any processes not accounted for in Control parameters. Thus, the Automatic parameters of the PDP can reasonably be conceptualized to reflect a combination of the Associations, Overcoming Bias, and Guessing parameters of the Quad model. As the present research indicates, Overcoming Bias and Guessing demonstrate unambiguously poor recoverability; consequently, their influence “contaminates” the Automatic parameters. From this perspective, Associations parameters in the Quad model would seem to be a purer index than Automatic parameters in the PDP of the strength with which a target category is associated with an attribute. Thus, both psychometrically and theoretically, Associations parameters may be better candidates for assessing and predicting individual differences than Automatic parameters—but both exhibit only moderate reliability for individual-level inference. Though, to be clear, we make no claims that any MPT parameter is a pure measure of any process or construct. Instead, we interpret MPT parameters to be relatively more process-pure than summary statistics (e.g., D-scores), and recognize that different parameters can vary in their process purity.

### Practical Implications for Prediction

In addition to illuminating the qualitative nature of the processes that underlie responses on implicit measures, the present research is also useful for researchers who apply formal models to their own work. Researchers often seek to correlate model parameters with theoretically-relevant individual differences measures (e.g., behavior, self-report). The reliability with which a variable can be measured imposes an upper limit on the extent to which the association between any two variables can be observed (Kanyongo et al., 2007; Loken & Gelman, 2017; Nunnally, 1970; Schmidt & Hunter, 1996; Spearman, 1904). Specifically, the correlation between two measures is constrained by each measure’s reliability, and thus analyses will have less statistical power if one or more variables is measured unreliably. Consequently, the Detection and Control parameters would seem to be the most promising candidates to correlate with individual difference measures because of their fair consistency between measurement occasion and acceptable recoverability within measurement occasion. Associations and Automatic parameters may be candidates to correlate with individual differences, but the extent to which their poor consistency reflects changes in “true” scores versus measurement error is unclear given their only modest parameter recoverability.

Though Associations, Automatic, Overcoming Bias, and Guessing parameters did not demonstrate acceptable recoverability in the present research, they may still be useful in some research contexts. For example, given that less reliable measures require larger samples to detect effects, researchers who are interested in the constructs reflected in the Associations and Automatic parameters would be well-suited to rely on large datasets, such as the ones available from Project Implicit. In fact, despite low-to-moderate meta-analytic recovery estimates for these parameters, their recoverability estimates in the much larger Project Implicit datasets were generally strong: Association parameter recovery ranged .78 to .83, and Automatic parameter recovery ranged .63 to .70. One potential interpretation of this pattern of results is that the large samples enabled the hierarchical Bayesian estimation method to produce more accurate population estimates, which in turn produced more reliable individual-level estimates. With that said, even the more recoverable Association and Automatic parameters estimated from the Project Implicit data demonstrated poor consistency across measurement occasion (ranging .10–.14, .26–.28, respectively), which suggests that these parameters are context-dependent rather than stable but noisily measured—but future research will need to continue to investigate this point. Moreover, Overcoming Bias and Guessing recoverability estimates were poor, despite the large samples. Nevertheless, model parameters with low measurement reliability can still be robust predictors at the group level (Hedge et al., 2018). Thus, the unequivocally poor recoverability and consistency of the Overcoming Bias parameter suggest that it is not viable for individual-level inference, but the possibility remains that its population-level estimates may be validly examined in the context of group-level inference. Finally, Guessing demonstrated very poor psychometrics, with consistency that includes zero, so we caution against any strong interpretations of Guessing, at either the individual or group level. Nonetheless, Guessing may still have value in model-based analyses: Guessing is configured to reflect a “catch-all,” accounting for residual variance not otherwise reflected in the other model parameters, which may in turn improve the precision with which other parameters are estimated (Wilson & Collins, 2019). Indeed, the value of the Guessing parameter may be illustrated by the descriptively greater reliability of the Association parameters in the Quad model than the Activation parameters in the PDP.

Our findings would also seem to dovetail with related lines of research aimed at characterizing and predicting mental states and behavior. For example, research in functional magnetic resonance imaging (fMRI) has devoted a great deal of research to understanding test–retest reliability, with significant implications for the clinical applications of fMRI as a tool for diagnosing biomarkers of mental health risk (Bennett & Miller, 2010; Elliott et al., 2020; Herting et al., 2018). In parallel, research on economic decision-making and reinforcement learning has also interrogated the test–retest reliability of computational parameters fit to behavior (Mkrtchian et al., 2022), with potential utility for understanding mental health and psychiatric symptoms. Mirroring this work, MPT parameters can only accurately assess individual differences in the processes that contribute to responses on implicit measures if the parameters can be measured reliably. Toward that end, the present research suggests that the Detection and Control parameters are sufficiently reliable to be used to predict individual differences.

### Interdisciplinary Implications for Cognitive Modeling

Given our reliance on MPT modeling, the present research is relevant to researchers across disciplines who rely on similar models rooted in the dual-process tradition of automaticity and control. Jacoby’s (1991) work to disentangle the contributions of recollection and familiarity to recognition memory inspired the PDP (Payne, 2001) as we applied it in the present research. This modeling approach has also been used to investigate a wide variety of topics, including executive functioning (Ito et al., 2015), evaluative conditioning (Hütter et al., 2012), judgment and decision-making (Ferreira et al., 2006), and moral reasoning (Conway & Gawronski, 2013). Our findings contribute to these literatures because, to our knowledge, little research has evaluated the retest reliability of MPT parameters (but see Luke & Gawronski, 2022). Similarly, our findings are relevant to researchers across disciplines who rely on response conflict-type measures like the IAT, which shares structural features with the Stroop task (Stroop, 1935), go/no-go task (Donders, 1969), and others. Models and measures like these are used across the cognitive sciences, and formal modeling offers a precisely-specified framework that can facilitate collaboration and theoretical advancement across disciplines (Calanchini et al., 2018). Consequently, the present research offers a roadmap for future investigations into the qualitative nature of a wide variety of cognitive processes.

### Limitations

The present research is limited in several ways. For example, MPT modeling relies solely on response accuracy, whereas the vast majority of IAT research is based on the D-score (Greenwald et al., 2003), which relies primarily on response latency. That said, accuracy- versus latency-based operationalizations of IAT compatibility effects often reveal the same pattern of results (e.g., Meissner & Rothermund, 2013). Nevertheless, future research should explore the generalizability of our findings with modeling approaches that rely solely on response latency (Haines et al., 2020), or incorporate both response latency and accuracy (Heck & Erdfelder, 2016; Klauer et al., 2007; Klauer & Kellen, 2018).

The present research is also limited in our reliance only on the race version of the IAT. Qualitatively different cognitive processes may contribute to responses on IATs configured to assess other constructs (e.g., sexism; homophobia; stereotypes; self-concepts). That said, the Detection and Overcoming Bias parameters of the Quad model operate similarly across IATs configured to assess different constructs (Calanchini et al., 2014), and the Control parameters of the PDP operate similarly across different implicit measures (Volpert-Esmond et al., 2020).

Relatedly, the present research is limited by its sole reliance on the IAT. The cognitive processes reflected in an implicit measure will vary depending on the structure and task demands of the measure (Payne et al., 2008). Thus, future research should investigate whether the pattern of results we report here generalizes to different constructs and measures. Finally, all of our inferences were largely based on relatively arbitrary criteria proposed by one set of researchers (Cicchetti & Sparrow, 1981), but other reasonable criteria have been proposed (Koo & Li, 2016).

## Conclusion

The present research used formal modeling to investigate the reliability and stability of the processes that contribute to responses on the race IAT. Replicating across two MPTs and six independent datasets, we found that accuracy-oriented processes can be reliably measured and are somewhat stable within individuals, but other processes are less reliably measured and may vary across measurement occasions. These findings advance implicit social cognitive theory by providing insight into the temporal stability of cognitive processes that contribute to responses on implicit measures, which highlights the processes that can be expected to predict behavior and other individual differences. In turn, this work offers a model-based template for future researchers to investigate the temporal stability of cognitive processes that may be overlooked by other analytic approaches.

## Supplemental Material

sj-docx-1-psp-10.1177_01461672231171256 – Supplemental material for Estimating the Reliability and Stability of Cognitive Processes Contributing to Responses on the Implicit Association Test Supplemental material, sj-docx-1-psp-10.1177_01461672231171256 for Estimating the Reliability and Stability of Cognitive Processes Contributing to Responses on the Implicit Association Test by Jacob Elder, Liz Wilson and Jimmy Calanchini in Personality and Social Psychology Bulletin

## Notes

1. In this manuscript, we use the term “implicit” to mean “indirect.” Thus, an “implicit measure” assesses mental contents indirectly, in contrast to other forms of measurement that assess mental contents through direct inquiry. Our use of this term contrasts with other definitions that refer to the qualitative nature of the construct (e.g., unconscious), but aligns with the perspective that implicit measures assess evaluations under suboptimal processing conditions (De Houwer & Boddez, 2022). Here, we specify indirect measurement as the defining procedural feature of the task that is the focus of the present research (i.e., the Implicit Association Test; Greenwald et al., 1998).

2. In this manuscript, we use the term “association” to refer to one of the mental constructs that influences responses on implicit measures. However, we adopt the term largely in recognition of its conventional use, but we make no strong assumptions about the representational nature—associative or otherwise—of the construct.

3. IAT attribute category labels were slightly different across datasets. The Project Implicit, Wilson and Calanchini, and Lai datasets used “good” and “bad” as category labels. Gawrsonki used “positive” and “negative” as category labels. Forscher used “pleasant” and “unpleasant” as category labels.

4. The parameter names “Automatic” and “Control” correspond to the dual-process perspective of cognition (e.g., Metcalfe & Mischel, 1999; Schneider & Shiffrin, 1977) in which the field of implicit social cognition is deeply rooted. Unfortunately, these terms have become linked over the years with assumptions about the awareness, intentionality, controllability, efficiency, and speed of processes described as automatic or controlled—assumptions that have not always been backed by empirical support (Melnikoff & Bargh, 2018; Moors & De Houwer, 2006). Consequently, we retain these labels to describe the two parameters of the PDP for linguistic convenience, but expressly make no assumptions about the qualitative nature (e.g., unawareness) of the cognitive process or processes that either parameter reflects beyond what the analyses reported in the present research can support.

5. The McGraw and Wong (1996) convention would be ICC(C,1).
