# Supplementary material for: Estimating the Reliability and Stability of Cognitive Processes Contributing to Responses on the Implicit Association Test
Source: Pers Soc Psychol Bull. 2023 May 19;50(10):1451–70. doi: 10.1177/01461672231171256 (PMC11367805; doi:10.1177/01461672231171256)
Supplement: sj-docx-1-psp-10.1177_01461672231171256 – Supplemental material for Estimating the Reliability and Stability of Cognitive Processes Contributing to Responses on the Implicit Association Test [file sj-docx-1-psp-10.1177_01461672231171256.docx]

Table 1. *Spearman Rho correlations depicting the similarity of simulated IAT responses for Quad model. Each column corresponds to a different response type for simulated response frequencies. Each row corresponds to a different study and measurement occasion. The bottom row and farthest right column reflect the average of all response frequencies, and the bottom-right cell depicts the average of all correlations across studies and measurement occasions. Top table reflects the similarity among simulations. Bottom table reflects the similarity between simulations and true behavior.*

| Quad Model Similarity Across Simulations | | | | | | | | | |
| --- | --- | --- | --- | --- | --- | --- | --- | --- | --- |
| *Study* | *1* | *2* | *3* | *4* | *5* | *6* | *7* | *8* | *Average* |
| Wilson & Calanchini, 2022 T1 | 0.093 | 0.135 | 0.103 | 0.136 | 0.176 | 0.136 | 0.272 | 0.250 | 0.163 |
| Wilson & Calanchini, 2022 T2 | 0.277 | 0.211 | 0.277 | 0.216 | 0.253 | 0.266 | 0.377 | 0.212 | 0.261 |
| Forscher et al., 2017 T1 | 0.231 | 0.256 | 0.272 | 0.264 | 0.309 | 0.272 | 0.353 | 0.254 | 0.276 |
| Forscher et al., 2017 T2 | 0.305 | 0.325 | 0.279 | 0.345 | 0.307 | 0.273 | 0.276 | 0.288 | 0.300 |
| Gawronski et al., 2017 T1 | 0.125 | 0.167 | 0.122 | 0.166 | 0.177 | 0.127 | 0.089 | 0.116 | 0.136 |
| Gawronski et al., 2017 T2 | 0.203 | 0.186 | 0.202 | 0.181 | 0.227 | 0.210 | 0.246 | 0.298 | 0.219 |
| Lai et al., 2016 (Study 1) T1 | 0.046 | 0.072 | 0.048 | 0.077 | 0.113 | 0.102 | 0.159 | 0.187 | 0.100 |
| Lai et al., 2016 (Study 1) T2 | 0.056 | 0.088 | 0.060 | 0.083 | 0.113 | 0.073 | 0.231 | 0.092 | 0.100 |
| Lai et al., 2016 (Study 2) T1 | 0.189 | 0.200 | 0.186 | 0.197 | 0.187 | 0.172 | 0.188 | 0.163 | 0.185 |
| Lai et al., 2016 (Study 2) T2 | 0.201 | 0.200 | 0.196 | 0.203 | 0.192 | 0.198 | 0.221 | 0.220 | 0.204 |
| Project Implicit, 2020 T1 | 0.400 | 0.422 | 0.402 | 0.420 | 0.493 | 0.463 | 0.494 | 0.495 | 0.448 |
| Project Implicit, 2020 T2 | 0.469 | 0.487 | 0.469 | 0.487 | 0.482 | 0.474 | 0.475 | 0.502 | 0.481 |
| Average | 0.216 | 0.229 | 0.218 | 0.231 | 0.252 | 0.231 | 0.282 | 0.256 | 0.239 |
|  | | | | | | | | | |
| Quad Model Similarity of Simulations to True Behavior | | | | | | | | | |
| *Study* | *1* | *2* | *3* | *4* | *5* | *6* | *7* | *8* | *Average* |
| Wilson & Calanchini, 2022 T1 | 0.140 | 0.202 | 0.157 | 0.195 | 0.256 | 0.227 | 0.434 | 0.353 | 0.246 |
| Wilson & Calanchini, 2022 T2 | 0.328 | 0.251 | 0.342 | 0.257 | 0.273 | 0.334 | 0.440 | 0.292 | 0.315 |
| Forscher et al., 2017 T1 | 0.298 | 0.273 | 0.314 | 0.327 | 0.447 | 0.262 | 0.486 | 0.340 | 0.343 |
| Forscher et al., 2017 T2 | 0.372 | 0.378 | 0.318 | 0.489 | 0.363 | 0.259 | 0.463 | 0.401 | 0.380 |
| Gawronski et al., 2017 T1 | 0.221 | 0.145 | 0.128 | 0.287 | 0.276 | 0.191 | 0.221 | 0.237 | 0.213 |
| Gawronski et al., 2017 T2 | 0.229 | 0.232 | 0.309 | 0.236 | 0.350 | 0.258 | 0.368 | 0.477 | 0.307 |
| Lai et al., 2016 (Study 1) T1 | 0.113 | 0.094 | 0.079 | 0.162 | 0.186 | 0.165 | 0.288 | 0.329 | 0.177 |
| Lai et al., 2016 (Study 1) T2 | 0.114 | 0.158 | 0.132 | 0.192 | 0.167 | 0.149 | 0.414 | 0.226 | 0.194 |
| Lai et al., 2016 (Study 2) T1 | 0.235 | 0.225 | 0.249 | 0.241 | 0.258 | 0.220 | 0.305 | 0.244 | 0.247 |
| Lai et al., 2016 (Study 2) T2 | 0.225 | 0.231 | 0.252 | 0.268 | 0.261 | 0.264 | 0.366 | 0.342 | 0.276 |
| Project Implicit, 2020 T1 | 0.450 | 0.459 | 0.439 | 0.491 | 0.544 | 0.506 | 0.552 | 0.551 | 0.499 |
| Project Implicit, 2020 T2 | 0.515 | 0.506 | 0.520 | 0.541 | 0.527 | 0.485 | 0.517 | 0.540 | 0.519 |
| Average | 0.270 | 0.263 | 0.270 | 0.307 | 0.326 | 0.277 | 0.404 | 0.361 | 0.310 |

Table 2. *Spearman Rho correlations depicting the similarity of simulated IAT responses for PDP. Each column corresponds to a different response type for simulated response frequencies. Each row corresponds to a different study and measurement occasion. The bottom row and farthest right column reflect the average of all response frequencies, and the bottom-right cell depicts the average of all correlations across studies and measurement occasions. Top table reflects the similarity among simulations. Bottom table reflects the similarity between simulations and true behavior.*

| PD Model Similarity Across Simulations | | | | | | | | | |
| --- | --- | --- | --- | --- | --- | --- | --- | --- | --- |
| *Study* | *1* | *2* | *3* | *4* | *5* | *6* | *7* | *8* | *Average* |
| Wilson & Calanchini, 2022 T1 | 0.093 | 0.135 | 0.103 | 0.136 | 0.176 | 0.136 | 0.272 | 0.250 | 0.163 |
| Wilson & Calanchini, 2022 T2 | 0.277 | 0.211 | 0.277 | 0.216 | 0.253 | 0.266 | 0.377 | 0.212 | 0.261 |
| Forscher et al., 2017 T1 | 0.231 | 0.256 | 0.272 | 0.264 | 0.309 | 0.272 | 0.353 | 0.254 | 0.276 |
| Forscher et al., 2017 T2 | 0.305 | 0.325 | 0.279 | 0.345 | 0.307 | 0.273 | 0.276 | 0.288 | 0.300 |
| Gawronski et al., 2017 T1 | 0.125 | 0.167 | 0.122 | 0.166 | 0.177 | 0.127 | 0.089 | 0.116 | 0.136 |
| Gawronski et al., 2017 T2 | 0.203 | 0.186 | 0.202 | 0.181 | 0.227 | 0.210 | 0.246 | 0.298 | 0.219 |
| Lai et al., 2016 (Study 1) T1 | 0.046 | 0.072 | 0.048 | 0.077 | 0.113 | 0.102 | 0.159 | 0.187 | 0.100 |
| Lai et al., 2016 (Study 1) T2 | 0.056 | 0.088 | 0.060 | 0.083 | 0.113 | 0.073 | 0.231 | 0.092 | 0.100 |
| Lai et al., 2016 (Study 2) T1 | 0.189 | 0.200 | 0.186 | 0.197 | 0.187 | 0.172 | 0.188 | 0.163 | 0.185 |
| Lai et al., 2016 (Study 2) T2 | 0.201 | 0.200 | 0.196 | 0.203 | 0.192 | 0.198 | 0.221 | 0.220 | 0.204 |
| Project Implicit, 2020 T1 | 0.400 | 0.422 | 0.402 | 0.420 | 0.493 | 0.463 | 0.494 | 0.495 | 0.448 |
| Project Implicit, 2020 T2 | 0.469 | 0.487 | 0.469 | 0.487 | 0.482 | 0.474 | 0.475 | 0.502 | 0.481 |
| Average | 0.216 | 0.229 | 0.218 | 0.231 | 0.252 | 0.231 | 0.282 | 0.256 | 0.239 |
|  | | | | | | | | | |
| PD Model Similarity of Simulations to True Behavior | | | | | | | | | |
| *Study* | *1* | *2* | *3* | *4* | *5* | *6* | *7* | *8* | *Average* |
| Wilson & Calanchini, 2022 T1 | 0.154 | 0.254 | 0.258 | 0.258 | 0.279 | 0.320 | 0.506 | 0.384 | 0.302 |
| Wilson & Calanchini, 2022 T2 | 0.392 | 0.313 | 0.447 | 0.368 | 0.437 | 0.490 | 0.466 | 0.368 | 0.410 |
| Forscher et al., 2017 T1 | 0.306 | 0.330 | 0.437 | 0.333 | 0.466 | 0.429 | 0.593 | 0.457 | 0.419 |
| Forscher et al., 2017 T2 | 0.260 | 0.390 | 0.400 | 0.557 | 0.247 | 0.281 | 0.532 | 0.497 | 0.395 |
| Gawronski et al., 2017 T1 | 0.306 | 0.248 | 0.163 | 0.280 | 0.284 | 0.308 | 0.251 | 0.312 | 0.269 |
| Gawronski et al., 2017 T2 | 0.262 | 0.232 | 0.320 | 0.212 | 0.395 | 0.272 | 0.421 | 0.374 | 0.311 |
| Lai et al., 2016 (Study 1) T1 | 0.162 | 0.042 | 0.120 | 0.224 | 0.287 | 0.089 | 0.306 | 0.429 | 0.208 |
| Lai et al., 2016 (Study 1) T2 | 0.226 | 0.161 | 0.243 | 0.231 | 0.234 | 0.190 | 0.407 | 0.254 | 0.243 |
| Lai et al., 2016 (Study 2) T1 | 0.322 | 0.224 | 0.291 | 0.270 | 0.280 | 0.248 | 0.352 | 0.293 | 0.285 |
| Lai et al., 2016 (Study 2) T2 | 0.311 | 0.263 | 0.255 | 0.281 | 0.287 | 0.381 | 0.361 | 0.356 | 0.312 |
| Project Implicit, 2020 T1 | 0.512 | 0.488 | 0.490 | 0.530 | 0.638 | 0.584 | 0.584 | 0.569 | 0.549 |
| Project Implicit, 2020 T2 | 0.544 | 0.547 | 0.574 | 0.589 | 0.617 | 0.566 | 0.571 | 0.566 | 0.572 |
| Average | 0.313 | 0.291 | 0.333 | 0.344 | 0.371 | 0.347 | 0.446 | 0.405 | 0.356 |

**Reliability of IAT Responses Across Simulations**

***Analysis Plan***

We performed 200 simulations of participant responses given their set of Quad model and PDP parameters. For both models, IAT responses are represented as a vector of 8 different response frequencies for each participant^^[[1]](#footnote-0)^^. We performed two separate tests for each model. First, we examined how similar the simulated response frequencies were with one another across all simulations. For each study, we extracted the *n*th response type (from 1 to 8) for all 200 simulations and, because these responses consist of non-parametric count data, we correlated them with each other using Spearman Rho. The resulting correlation matrix reflects the correlations for the *n*th response type across 200 simulations for study *i*. We estimated the mean of the lower triangle of the correlation matrix. We iterated this process across each response type and each study, generating a table of “similarity” estimates between simulated response frequencies of each response type and within each study. Finally, we estimated the mean of each response type’s similarity estimates within each study, as well as the mean of all studies’ similarities within each response type, in order to provide insight into how consistent response frequencies are simulated given each participant’s original MPT parameters.

As a second, complementary test, we examined how similar the simulated response frequencies were with participants’ true response frequencies. This test operated similarly to the first test, except that we iterated through studies and response types, then estimated the Spearman Rho correlation of each simulation’s response type *n* with the original study’s response type *n*. We calculated these correlations for all simulations within a study and response type, then averaged the correlations together for study *i* and count type *n*, iteratively across all studies and response types. This process produced a table of “similarity” estimates, which provides insight into how similar the response frequencies simulated from participants’ parameters were to participants’ original response frequencies.

**Time as a Moderator of Retest Reliability**

***Analysis Plan***

Approximate time intervals between measurement occasions are listed in Table 1, but we do not have precise information about measurement intervals for all datasets. Specifically, in the context of the Project Implicit dataset (which is the largest dataset by an order of magnitude), we know that participants completed both IATs within the same browser session, but have no information about how much time elapsed between measurements. Consequently, we assumed that browser sessions are relatively short on average, and ordinally ranked the datasets as follows for exploratory analysis:

1. Project Implicit, 2020

2. Wilson & Calanchini, 2022

3. Lai et al., 2016 (Study 1)

3. Lai et al., 2016 (Study 2)

4. Gawronski et al., 2017

5. Forscher et al., 2017

In this ranking, 1 reflects the shortest interval between measurement occasions (i.e., one browser session), and 5 reflects the longest interval between measurement occasions (i.e., 2 years). We treated measurement interval as an ordered categorical factor and modeled the interval moderator as an orthogonal polynomial contrast.

**Comparing Test-Rest Reliability and Parameter Recovery**

We investigated whether parameters differ in their within-measurement recoverability and between-measurement reliability.

***Analysis Plan***

We examined the extent to which recovery rates and retest reliability differed within each parameter by inspecting overlapping confidence intervals between test-retest ICCs and recovery correlations. These analyses provide exploratory insight into the extent to which a parameter’s recoverability aligns with its stability across time.

***Results***

The Detection and Black-bad Associations parameters of the Quad model are significantly more recoverable within measurement occasions than they are reliable across measurement occasions. However, the White-good Associations, Overcoming Bias, and Guessing parameters do not differ in their recoverability versus retest reliability.

All four Control parameters of the PDP, along with the Automatic-Black parameter, are significantly more recoverable within measurement occasions than they are reliable across measurement occasions. However, the Automatic-White parameter is more reliable across measurement occasions than it is recoverable within measurement occasions.

1. The IAT consists of 16 response categories: correct and incorrect responses to Black, White, good, and bad stimuli in compatible and incompatible blocks. However, incorrect responses are the complement to correct responses and, thus, they are redundant to one another. Therefore, we only modeled correct responses in this analysis. [↑](#footnote-ref-0)
